# Senile Respiratory Disorders

**Published:** 1907-12-07

**Authors:** R. Douglas Powell


### Hospital Clinics.

### SENILE RESPIRATORY DISORDERS.

By SIR R. DOUGLAS POWELL, Bt., K.C.V.O., M.D.

Abstract of a lecture delivered at the Medical Graduates' College and Polyclinic, November, 1907.

It is quite unnecessary for me to give you any definition of old age; you have probably had several already in the series of lectures which I am here to-day to continue. The fall of a leaf and the death of a tree are natural, healthy developmental processes; and so is old age a healthy process, not a disease. We have to speak of those respiratory diseases which tend to interrupt this natural decline of old age. It would be impossible to describe them at any length. I can only glance at them, knowing perfectly well that your experience will fill in the details.

### Emphysema.

Calcification of the cartilages of the body is a common event in old age. In those of the ribs it is especially seen, though Professor Humphry found several very old people whose cartilages remained perfectly soft. The calcareous degeneration may affect also the cartilages of the trachea and bronchi. Emphysema, too, is a frequent condition in old age, though, again, it is not an essential one. Now, calcification of the cartilages and senile emphysema are very important, because they interfere with the mechanisms of respiration and circulation.

Up to past middle life there is an elastic traction between the ribs and the pleura, which acts as a constant aspiratory force towards the chest cavity, favouring the return of blood towards the heart. The cardiac action is forcible enough easily to overcome this aspiratory suction and to propel the blood into the circulation: but the negative pressure within the chest is a very important factor in helping the venous return. When senile emphysema is fairly complete this force is lost: the ribs are no longer elastic and the lung itself is no longer contractile. In thin old people the jugular veins are frequently seen somewhat full, and becoming fuller in expiration; not very full, but more so than is normal in younger people. These changes are of little consequence in the quiescent life of the aged, because there is a lessened blood volume. Hence so long as they are not "hustled" to catch a train or to hurry up a hill, they do not suffer; but if they attempt such things they readily become distressed. Senile emphysema *per se* is not to be treated, but only to be remembered among the limitations of health.

### Quiescent Tuberculosis.

A localised fibrosis is often met with at one apex, generally the result of an old tubercular invasion. In a considerable number of old people who are examined post mortem there will be found on the surface of the lung a puckered fissure; this depression corresponds to destruction of the tissue beneath. It is important to bear in mind that these lesions are sometimes the cause of an altered breath sound, a conducted voice sound, and a little dry, creaking crepitation, the latter produced in the small emphysematous blebs of lung around the fibrosed tissue. Even in advanced life there sometimes occurs a sudden activity in an old focus of this sort, with hæmoptysis or other evidence of tuberculosis.

There is also a condition which used to be known as a "dilatation of a bronchus" at one apex, in the old, but I have not the slightest doubt that the signs which led to this diagnosis are really caused by old tuberculous nodules with central smooth wall excavation. In such cases sometimes there is a considerable amount of mucous expectoration, and you may not uncommonly find in it tubercle bacilli. Do not conclude that this is a proof of an active tuberculosis. I remember the case of a well-known member of Parliament—and a brilliant speaker—who for years had morning expectoration which contained tubercle bacilli. That man lived to a ripe age, nearer 80 than 70, and he kept up his parliamentary activity to the end.

### Basal Fibrosis.

Another condition is a kind of emphysema which it is very important to bear in mind. After an attack of pneumonia, bronchitis, or influenza, a permanent change is frequently, in elderly people, left behind at one base; some lack of resonance, or a higher pitch of percussion note, and a moderate quantity of fine, crepitant râle, somewhat variable but persistent, will be found. Such patients have an habitual cough and bring up a certain amount of clear mucous expectoration, in some cases more or less pigmented. At times they get an added bronchitis, or a catarrh of the upper air-passages, and the expectoration due to that is of a mucopurulent quality. They are short of breath and are liable to bronchitis. The point is that the local condition at the base does not clear up: it is one of chronic fibrosis, in the interstices of which is a greater or less degree of permanent œdema. The importance of recognising this state lies in the fact that it may be mistaken for a more active process. If the case has been watched all through, there need be no anxiety such as a doctor might feel were he examining it for the first time. There is a tendency to stagnation of the circulation at that base, with a danger of a more acute process setting in.

The treatment of this form of lung disablement is on general lines. You would not give expectorants, nor would you attempt to cause absorption by ordering potassium iodide. A little dilute acid with tincture of nux vomica is valuable to tone up the vessels, and may be given in some carminative and digestive mixture. The patient should be allowed exercise in the better part of the day, but a certain amount of rest must also be insisted on, and this is best obtained by one or two hours' complete quietude before getting ready for dinner. We must enjoin also light meals eaten slowly and leisurely with a small amount of wine. The functions of the liver must be carefully attended to by an occasional dose of blue pill or calomel followed by a morning saline. When the pulse is rapid or feeble small doses of digitalis are useful.

Climate is not unimportant, and the South Coast resorts often do good, such as St. Leonards, Falmouth, Weymouth, Bournemouth, Isle of Wight, and Torquay. Of foreign climates those of Egypt, Mentone, and Madeira are the best for these patients.

Strychnine.

I wish here to sound a note of caution as to the administration of strychnine to old people, which, although of great value, is, I am quite sure, sometimes overdone. Strychnine undoubtedly increases arterial tension, and excites old people to try more than they have strength for. Any course of this drug should, therefore, be not too prolonged, and small doses only should be ordered, such as two or three minims of the liquor strychninæ, or five to seven of the tincture of nux vomica.

Latent Inflammations.

Other troubles in aged people are latent pleurisy and latent pneumonia, which are not uncommon. These are not infrequently the "end-diseases"—the precursors of death. A latent pneumonia is one commencing in a stagnation or passive congestion at the base of the lung. When pleurisy is associated with it there may be pain, but pain in these latent cases is often referred to other regions, such as the abdomen, and may be confused with that of colic. Often there is no very marked acceleration of breathing or of the pulse to attract attention, and it is very easy indeed to overlook the real disease, and to treat the abdominal symptoms only.

The symptoms of onset of latent pneumonia are fatigue and exhaustion, with very little cough and very little expectoration. Occasionally there is a sudden collapse, with failure of the heart. Like young infants, old people are apt to die suddenly after exposure, and the lesions found are generally those of acute pneumonia or of acute bronchitis. Accident or shock is sometimes the exciting cause of this kind of pneumonia. There is always some increase of the pulse-rate, but as the normal pulse in old age is slow, the actual count may not seem a rapid one. Respiration is quickened comparatively more than is the pulse. The condition is generally fatal, but the best chance of recovery is afforded by the use of stimulants and complete rest and warmth. Warmed inhalations of oxygen are of great value, a point to which I shall again refer.

Cheyne-Stokes Respiration.

There is another condition—I am not sure that I can really call it a respiratory disease—which is not rare in old people, and that is the condition known as Cheyne-Stokes respiration. This was first described nearly a century ago (1818) by Cheyne, of Dublin, and his description was elaborated many years later by Stokes (1837). It is often induced by over-fatigue or over-excitement. The respiratory movements become more and more shallow, to complete cessation lasting for 10 or 15, even for 40, seconds, followed by rapidly deepening inspiratory efforts. When these have reached their maximum the mouth is often open, with slight tugging downwards of the angles. There is hardly any nasal action; the breathing is almost always oral. The pulse rhythm may be a disorderly one, but *it is not altered during the respiratory pause.* Lividity is often much less than one would expect. Towards the end of the pause there is sometimes twitching of the corners of the mouth and of the extremities. During sleep you may often see Cheyne-Stokes breathing in old folk, and when attacks occur in the day, consciousness is very geenrally somewhat diminished, though the patient need not be seriously ill.

This phenomenon is a common and grave symptom in many diseases. In uræmia, fatty degeneration of the heart, and cardio-vascular sclerosis, it is a frequent end-symptom. The mechanism is not fully made out, but it is supposed to be due to defective innervation of the pneumogastric centre in the bulb, which in consequence does not respond with sufficient quickness to the natural stimulus of venous blood. But the absence of lividity is against this hypothesis, and it is possible that the cause is an exhaustion of the centre, which requires a longer rest than it can get in the ordinary way of respiration. The process also recalls somewhat those cardiac cases in which several auricular contractions take place before the impulse can travel along the bundle of His and stimulate the ventricle to contraction.

The remedies indicated are strychnine and caffeine, which often meet with a very notable degree of success. I remember an old man of 75 or 77, the subject of cardiac degeneration, who had a most marked degree of Cheyne-Stokes respiration. He was very anxious to live to see his daughter's marriage, which was to take place in two months' time; by frequent oxygen inhalations and by strychnine and caffeine he actually lived for two years.

Oxygen Inhalations.

Of the value of oxygen I should like to say a word, because it is very great. In senile pneumonia, in senile bronchitis, in senile Cheyne-Stokes breathing, and in all forms of senile heart disease, it is a most valuable stimulant, often far more so than alcohol. The rationale of this is that it leads to a supply of freshly-oxygenated blood to the cardiac muscle. No doubt the pneumogastric centres also benefit by the better-arterialised blood they receive. The usefulness of this remedy for the fatigue of old people is also marked: a brief inhalation of oxygen often does more than an alcoholic stimulant.

It is important to give the oxygen warmer than it issues from the cylinder. I always advise the intervention of an elastic bag of considerable size to take off the chill of expansion. In some cases it is desirable to warm the gas still more by a wash-bottle of hot water, which also slightly moistens it. Another useful method is to pass the gas through a metal tube warmed by a hot-water chamber. (Apparatus shown as supplied by Messrs. Krohne and Sesemann.

Bronchitis.

Asthenic bronchitis is one of the most fatal diseases of old people. In it, of course, one of the elements of mortality is the feeble power of expansion due to rigid ribs and emphysema. The malady is very fatal also, as you know, at the beginning of life, and in both the young and old its course is often extremely rapid. I will relate to you the case of a widow, aged 76, who was dining with friends on December 27 in her usual good health and spirits. On the way home she was supposed to have been exposed to a chill. Next day she did not feel very well, and suddenly late at night she became severely ill and was found in a state of collapse. No morbid sounds could be heard in her lungs except a few rhonchi posteriorly. Free stimulation rallied her so much that her friends became hopeful. By the following mid-day the chest-signs were more marked, however, with short inspirations and prolonged wheezy expirations. Cough was now frequent and difficult, and expectoration profuse. The pulse and temperature were 90 and 100° F. respectively: there was slight delirium. Forty-eight hours after the onset fine bubbling râles were audible, most abundant at the posterior bases, where the air entry was very poor. There was the characteristic breathing of considerable emphysema; and a tracheal rattle, at first removable by cough, set in. Death closed the scene at about the 70th hour.

The characteristic features of this case are the profound shock, almost producing a fatal result as early as the twelfth hour (quite a number of people die in this stage); then the rally and the onset of slight febrile reaction and bronchitic signs, followed on the third day by the peculiar short inspirations, prolonged laboured expiration and trachial râle which usher in the fatal result; recovery is indeed rare when once this stage has been reached. The common causes of bronchitis in old people are shock, chill, or the contagion of a common cold caught from someone else.

It is not necessary to say much about winter bronchitis. I have already explained the tendency to bronchitis in old people, and its association with chill. With regard to treatment, you must maintain the temperature of the room at 65° F., taking care also to have it well ventilated. I would utter a warning against the practice of wrapping the chests of old people in poultices, which add greatly to the fatigue of their respiratory movements. The occasional application of linseed or mustard poultices is of value, followed by pine- or cotton-wool applications, especially during the night.

The danger of bronchitis is from the exhaustion and paralysis of the bronchial tubes. Do not oppress your patients with too much food, give small quantities at regular intervals so as not to overload the stomach. Ammonia, digitalis, strychnine have their place, but do not give the first and the last together in the same bottle. Senega and serpentary are useful as the bronchitis wanes, but in the early acute stages ipecacuanha and potassium iodide are better.

In chronic winter cough with copious expectoration tar preparations are very good. Two especially I wish to mention. One is Eau de Goudron, which in Vichy or Ems water I have often found valuable in cases of bronchorrhœa. The other is liquor picis aromaticus (Bell), which contains about seven and a half grains of tar to the drachm. A good prescription which hides the nauseous taste of the tar, and is also stimulative and carminative, is:—

| | | |
|---|---|---|
| ℞ Liq. Picis Aromat. (Bell) | ... ... | ʒij. |
| Glycerini | ... ... ... ... ... | ʒiv. |
| Ext. Liq. Glycyrrhizæ | ... ... ... | ʒiss. |
| Sp. Ammon. Aromat. | ... ... ... | ʒiv. to ʒvj |
| Tr. Card. Co. | ... ... ... ... | ʒiv. to ʒvj |
| Aq. Chloroformi | ... ... ... ad | ℥viij. |

Sig: A sixth part thrice a day.

I need not add that climatic treatment is of value.

With regard to acute pneumonia, the prognosis is much less grave than is that of acute bronchitis. Quite old people whose senses are somewhat blunted will often recover from this condition, whereas ten years previously they would have succumbed.

